# Supplementary material for: Exceptional preservation and foot structure reveal ecological transitions and lifestyles of early theropod flyers
Source: Nat Commun. 2022 Dec 20;13:7684. doi: 10.1038/s41467-022-35039-1 (PMC9768147; doi:10.1038/s41467-022-35039-1)
Supplement: Supplementary file 1 — Supplementary Information [file 41467_2022_35039_MOESM1_ESM.pdf]

Exceptional Preservation and Foot Structure Reveal Ecological Transitions and Lifestyles of Early Theropod Flyers

This Supplementary Information file consists of Supplementary Figure 1, Supplementary Descriptions of Early Theropod Flyer Podothecae, Supplementary Descriptions of Extreme Points in PCA, Supplementary Information on Tarsometatarsus Length as a Variable for Traditional Morphometrics and Supplementary References.

Supplementary Figure

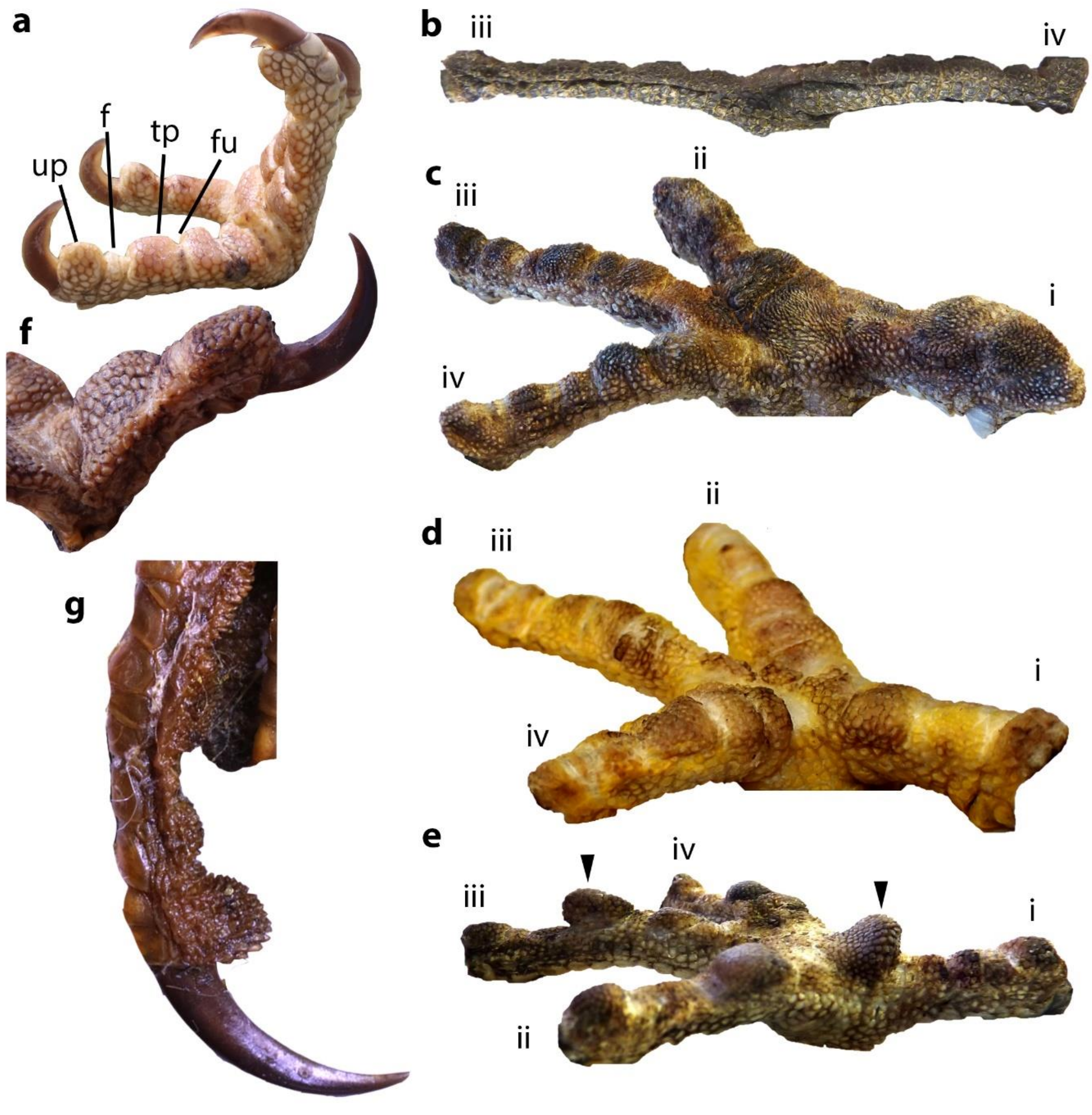

Supplementary Figure 1. Modern avian podothecae. A, Zygodactyl foot of the Musk Lorikeet (*Glossopsitta concinna*); B, Galah (*Eolophus rosiecapillus*) showing flat toe pads; C, White Bellied Sea Eagle (*Haliaeetus leucogaster*) showing well developed toe pads and sharp spiculate scales; D, Black-shouldered Kite (*Elanus axillaris*) showing well developed toe pads with smooth, globose reticulate scales; E, Brown Falcon (*Falco berigora*) showing well developed and protrustional (arrowheads) toe pads; F, Smooth, globose reticulate scales in the Pacific Baza (*Aviceda subcristata*); G, Spiculate scales in the Whistling Kite (*Haliastur*

sphenurus). Images not to scale. Abbreviations: i–iv, digits i–iv; f, fold; fu, furrow; tp, toe pad; up, ungual (claw) pad. Figure parts B-E were produced from the same original specimen photos that were used to produce Figure 4 of Tsang *et al.* [1].

Supplementary Descriptions of Early Theropod Flyer Podothecae

Microraptor

STM 5-109

This specimen includes a near-complete, semi-articulated right pes, exposed in medial or ventromedial view. The bones of the pes are three-dimensional, with only minor crushing of some elements. Metatarsal I and the phalanges of the hallux (digit I) are semi-articulated and lie adjacent to and separated from metatarsal II. Phalanges II-2 and II-3, and III-2 and III-3, and all phalanges of digit IV are articulated, while the remaining phalanges of digits II and III have shifted slightly from their life position. Digits III and IV are completely separated; however, superimposition of the most proximal phalanges of these digits (as well as between digits II and III) obscure further details.

Under white light, soft tissues appear as a relatively homogenous white silhouette that surrounds parts of the foot skeleton, most distinctly around digit IV. Individual scales or scale patterns are poorly discernible under white light. However, under laser-stimulated fluorescence, soft tissues of the podotheca are visible as a bright yellow ‘film’, which enshrouds nearly the entire pes and partly overlies several phalanges. Digital pads are arthrally-arranged and well-defined, separated by variably-sized interpad furrows. Digit III preserves three well-developed pads (including the claw pad) with a broad fold between the first and second pad. The claw pad is the lowest of the three pads, is incompletely preserved and separated by a broad furrow from the second toe pad. Digit IV preserves four pads, including the claw pad. The first pad (spanning the joint between phalanges IV-1 and IV-2) is protrusive, semi-oval in outline, and separated from the second pad by a broad fold. A furrow separates the second and third toe pad. The first pad is widely separated from the tarsal region by a wide furrow—the widest anywhere on the foot—although the tarsal pad itself is not preserved. Besides the claw sheath (see below), the integument of digit II is not visible.

Three distinct scale morphologies are present: 1) sub-rectangular, polarised, scutate scales (>1.2 mm high by ~0.5 mm proximodistally long) that tightly abut, do not imbricate, and are aligned in a singular arching row dorsal to phalanges III-2, IV-3 and IV-4; 2) polygonal, subrounded and irregular, non-imbricating scutellate scales (~0.5 mm diameter) that cover the dorsolateral surfaces of the digital pads, and the lateral surfaces of phalanges II-2, III-3 and III-4, and IV-1, 3, 4, and; 3) minute polygonal, subrounded and irregular, non-imbricating reticulate scales (~0.2 mm diameter) that cover undersides of the digital pads. Where exposed in lateral view (i.e., along the ventral margins), the reticulae on the undersides of the pads are sometimes sharp and ventrally protruding, forming distinctive spicules. These are most apparent on the second pad of digit IV.

The transitions between each of the scale types (scutate, scutellate, reticulate) are relatively abrupt. Although scutate scales are only preserved on the dorsal surfaces of three phalanges, it is expected that these extended the entire length of each digit (excluding all but the proximal-most part of the claw). There is, however, no indication of any scales on the metatarsus, which instead preserves elongate feathers in other known *Microraptor* specimens [2,3].

The hypertrophied digit II claw preserves a complete keratin sheath that is 136.5% longer than the claw itself. Incomplete keratin sheaths are also visible on the claws of digits

III and IV. In all instances, these keratin sheaths are preserved as a thin (<1 mm thick) brown film.

STM 5-75
The complete articulated left pes of STM 5-75 is exposed in lateral view. Digit II is hyperextended whereas digits III and IV are relatively straight or gently flexed, respectively, clearly exposing the soft tissues on the underside of each digit. Digit I is not visible. Under white light, there is almost no evidence of soft tissues associated with the podotheca; however, under LSF, they are revealed on all three main digits as well as ventral to the distal tarsals. In general, the soft tissues are not as well preserved as those in STM 5-109 and only scutate and reticulate scales could be identified. Scutate scales are only visible in a small area dorsal to phalanx II-2, where they are subrectangular (mediolaterally widest in life) and exposed in lateral view. Subcircular or weakly polygonal reticulate scales occur on the undersides of the digital pads, the tarsal pad, and extending along the posterior aspect of the metatarsus for approximately one third of its length. Spicules are not apparent in any of these regions, although this may be a symptom of the relatively poor preservation of the specimen. Digital pads are arthrally arranged. Only the claw pad is preserved on digit II; it is only partly visible (due to foot orientation and consolidants used on the specimen) but appears well developed. On digit III, at least three well-developed pads are visible (or partly visible), separated by folds, although details between the distal tarsals and phalanx III-1 are obscured by the orientation of the pes. Digit IV preserves three well-developed proximal pads (the claw pad is not preserved) separated by furrows. Unlike in STM 5-109, the proximal-most pad of digit IV is also well-developed (not protrusive), but is separated from a deep, protrusive tarsal pad by a broad furrow. Complete, strongly-curved claw sheaths are preserved on digits II–IV, the largest of which is found on digit II.

Anchiornis

STM 0-1
Both articulated and complete pedes of STM 0-1 are preserved; the left pes is exposed in ventromedial view, whereas the right pes is exposed in ventrolateral aspect. The digits of both pedes are extended roughly parallel with the long axis of their respective metatarsus, and digits II and III are overlapping on both pedes. At least six soft tissue digital pads are wholly discernible within the feet of STM 0-1. Soft tissues of the left pes are the best preserved and show four well-developed, arthrally-arranged pads on digit IV. Although well-developed, they are semi-lenticular in outline and subsequently appear lower than in *Microraptor*. A fold is present between the penultimate pad and the claw pad, whereas furrows separate the remaining pads. Claw pads are partially preserved on digits II and III (the latter is also preserved on the right pes). The claw pad on digit III on the right pes, which is the best preserved, is more semi-circular in outline and consequently more protrusive than the other digital pads. Reticulate scales are best seen on digit IV of the left pes, where they are ~0.2 mm in diameter, subrounded, and uniformly distributed. Poorly preserved scales on the dorsal surface of digit III appear somewhat larger than the reticulate scales on digit IV and may represent scutellate scales. Their morphology, however, is indistinct owing the suboptimal preservation of the specimen.

STM 0-7
The right pes of STM 0-7 is articulated and exposed in ventromedial view. Each of the digits are roughly parallel to one another and the main axis of the metatarsus. Digits II and III are

partly overlapping, and the claw of digit III is missing. At least five soft tissue pads are wholly visible within the left pes under LSF. A single well-developed arthral pad is present on digit III between the second and third phalanx. The claw pad and two more proximal pads (that between phalanges 3 and 4 is not preserved) are associated with digit IV. The pads are well developed, semi-lenticular in outline, and, as in *Microraptor*, the proximal-most pad is the deepest. Folds and furrows cannot be distinguished due to the relatively poor preservation of the soft tissues. A single pad appears to be associated with the hallux although we cannot discount the fact that this may represent the tarsal pad, separated by an apparently wide furrow from the proximal pad of digit IV. Each pad is made up of minute (~0.2–0.4 mm diameter) subrounded reticulate scales, which have a relatively uniform arrangement. Additional scale types are not discernible.

STM 0-114

The left pes of STM 0-114 is complete and articulated with strongly flexed proximal phalanges, and is exposed in medial view. The penultimate phalanges, however, of both digits II and III are unnaturally hyperextended. Soft tissues are visible as a white ‘halo’ surrounding parts of each digit under white light. Under LSF, soft tissues are visible on the underside of all three main digits and the hallux, including the claw sheaths. Digit II is the most poorly preserved, and only the large, well-developed claw pad is visible. Digit III preserves two well-developed pads: the claw pad, which is the largest and best preserved in the specimen, and another spanning the joint between the first and second phalanx. The intervening pad between phalanges two and three is not visible, presumably due to stretching of the integument over the hyperextended articulation between these two phalanges. The three distal toe pads of digit IV are preserved, including the claw pad, although their outlines are not as defined and appear lower than those on digit III. As in STM 0-1, a fold is present proximal to the claw pad. The hallux preserves a well-developed claw pad and two smaller pads along the length of the first phalanx. Therefore, both arthral and mesarthral pads appear to be represented on the hallux. All digital pads, including those from the hallux, show minute reticulate scales (~0.2–0.4 mm diameter), which are generally subrounded in plan view. On the ventral margins of the pads on digits II–IV, where the reticulate scales are in lateral aspect, they form short (up to ~0.4 mm long) spiculate projections. In addition to those of the left pes, some scales are also visible along the anterior surface of the right ankle. These are similarly subrounded to the reticulate scales of the pedal digits, albeit slightly larger (~0.5 mm diameter) and less densely arranged. Sharply curved claw sheaths are present on all four of the digits. That on digit II, which is also the largest, is evidently misaligned with the claw itself; the distal tip of the claw apparently pierces the dorsal margin on the keratinous sheath.

STM 0-125

The complete articulated left pes of STM 0-125 is preserved in lateral aspect. Digits II-IV are extended parallel to the long axis of the metatarsus and are largely overlapping. Digit IV is partly separated from the other digits and is missing the claw phalanx. Consequently, the soft tissues are only interpretable along the plantar surface of digit IV and the distal end of digit III, although the claw sheaths are typically not discernible. A brightly fluorescent region ventral to the tip of phalanx II-3 may be a portion of that keratinous sheath. Three arthral, equal-sized and well-developed proximal toe pads separated by creases are present on digit IV; the claw pad is not clearly discernible, although reticulate scales are still preserved in this region. All toe pads have a low parabolic outline (semi-lenticular in shape). Soft tissues associated with digits I–III do not show clear pad morphology, although a fluorescent area in the region of the claw pad of digit III may be the poorly preserved remains of that pad. Each digit IV pad preserves minute reticulate scales (~0.3 mm diameter), which are subrounded,

and arranged in a relatively uniform mosaic. No other scale types or interpretable soft tissue are present on the podotheca.

STM 0-144

The complete articulated right pes of STM 0-144 is preserved in medial or ventromedial aspect. Digits II-IV are extended parallel to the long axis of the metatarsus and are largely overlapping, whereas digit IV is partly separated from the other digits. Phalanx IV-4 is truncated by a break in the rock, and the claw phalanx is subsequently missing. Many of the bones of the foot and elsewhere on the specimen are split longitudinally (either partially or completely) along the bedding plane, and are therefore partially preserved on both the slab and counterslab. Soft tissues of the podotheca are visible under white light as a faint, pale 'halo' surrounding the pes, with weak definition of the pads associated with digit IV and the tarsal pad. Under LSF, three arthrally-arranged, well-developed digital pads separated by creases are wholly discernible on digit IV; the last pad (the claw pad) is not preserved. The proximal-most pad of digit IV (between the first and second phalanx) is separated from the similarly well-developed tarsal pad by a wide furrow. Two additional digital pads are partly discernible, in the region of digits II and III, but are difficult to assign to one or the other owing to the overlapping of the digits and suboptimal preservation of the bones. All toe pads, including the tarsal pad, have a low parabolic outline (semi-lenticular in shape). Reticulate scales on the digital pads are subrounded in plan view (~0.1–0.35 mm diameter). Within the pads of digit IV, specifically, the larger reticulate scales are positioned dorsally and gradually reduce in size ventrally. Only a thin fluorescent band of soft tissue is visible on the dorsal part of digit IV and ?II, but individual scales are not discernible.

STM 0-147

The right pes of STM 0-147 is near-complete and articulated, exposed in lateral view. Digit IV is extended and gently flexed relative to the metatarsus, whereas digits II and III are overlapping, with the penultimate phalanges of both digits hyperextended. Soft tissues appear as a white silhouette surrounding the entire pedal skeleton of STM 0-147 under white light. Under LSF, all four arthrally-arranged digital pads of digit IV are present. These are well-developed (semi-lenticular in outline), separated by shallow furrows, except between the first and second pad, which are separated by a fold. The first digital pad of digit IV is separated from the equally well-developed tarsal pad by a wide furrow, although part of the furrow is damaged. Portions of additional digital pads from digit II and III are visible, but are not wholly discernible due to the partial superposition of these two digits. Reticulate scales cover the ventral surfaces of the tarsal and digital pads. These are subrounded in plan view (~0.14–0.56 mm in diameter), and are arranged in a relatively uniform mosaic. Spicules are not apparent. Scutellate scales cover the lateral and dorsolateral parts of the digits (~0.19–0.83 mm diameter), and are subrounded, polygonal or irregular in shape. Scutate scales appear present dorsal to the distal half of II-2 and possibly phalanx III-1, although the former may also pertain to digit III; overlapping of the digits makes this association difficult to distinguish. Where they are best preserved, dorsal to phalanx II-2, the scales themselves form a single faint row of rectangles (that would have been mediolaterally oriented in life).

The near-complete, articulated left pes is also preserved, but is in relatively poor condition; the claw sheath is present on the hallux, with less complete sheaths on digits III and IV. Of the remaining soft tissues of the podotheca, only the tarsal pad is preserved, which does not differ substantially from that on the right foot.

Sapeornis

41HIII0405

Both pedes of *Sapeornis* 41HIII0405 are articulated along with the entirety of the skeleton[4]. Both pedes are uncrushed, with well-defined scales and contours of the podotheca that are three-dimensionally preserved. The left pes, exposed in lateral view, shows the rear-facing and enlarged hallux (digit I), which is well separated from digits II-IV. The proximal phalanges of digits III and IV are overlapping, but the soft tissues of all three main digits (II–IV) also show some overlap. The right pes is exposed in plantar view (the claws and terminal phalanx of digits II–IV are rotated in medio-plantar view). Digits II–IV are splayed slightly, with less overlap than the left foot, although the soft tissues of the hallux and digit IV are partly overlapping. Subsequently, the left and right pedes provide a complementary record of the entire podotheca.

In addition to three-dimensional scales, the dark colour and high sedimentological contrast of the preserved soft tissues in 41HIII0405 further distinguish it from other paravian specimens described herein, which are typically compressed flat, and have poor contrast with respect to the surrounding matrix (under white light). These differences suggest a distinct preservation style in 41HIII0405. Furthermore, rounded to irregular scales across the podothecae in 41HIII0405 are morphologically comparable regardless of location, only differing in size (ranging ~0.15–0.72 mm in diameter). Thus, although this scale type occurs beyond the plantar surfaces of the feet, we collectively refer to these as reticulate scales, in the absence of distinct scutellate or scutate scales.

Yanornis

STM 9-531

The complete, articulated right pes of STM 9-531 is split between the part and counterpart. It is exposed in dorsal view with the digits extended, separated (not overlapping), and gently flexed relative to the metatarsus. Under white light, soft tissues of the entire podotheca surrounding the pes, as well as covering the bones themselves, are clearly visible. Under LSF, these details, particularly the definition of the individual scales, are enhanced. Shallow invaginations along the length of digit III, in-line with the joint between phalanx III-1 and III-2, and between phalanx III-2 and III-3, belie the position of the pads. The distal two pads of digit III, therefore, have an apparently mesarthral arrangement. On digit II, invaginations of the soft tissue adjacent to the middle of phalanx II-I indicate the presence of at least one pad with arthral arrangement, contrasting with the mesarthral pads on digit III. Similar pad invaginations are not apparent elsewhere on digits II and IV. Given the orientation of the specimen, reticulate scales cannot be seen. The subcircular scales that cover the majority of the pes, are interpreted as scutellate scales, are relatively large (0.5 mm diameter), and have similarly wide areas of interstitial tissue. Alternating dark and pale bands on the dorsal surface of the phalanges and distal metatarsus are interpreted as singular rows of scutate scales. Those on the metatarsus are broader than those on the digits, spanning metatarsals II–IV. The dark and pale bands are transversely broad and of consistent proximodistal length on digits II and III (pale and dark bands both $\approx$ 0.8 mm), whereas the proximodistal lengths differ on digit IV (pale bands $<$ 0.3 mm; dark bands $\approx$ 0.9 mm) and the distal metatarsus (pale bands $\approx$ 0.8 mm; dark bands $\approx$ 0.3 mm). Individual scales (including scutellate and scutate forms) are not as well preserved on the metatarsus as elsewhere, although it seems that the metatarsus probably bore scales for approximately three-quarters of its length; the proximal one-quarter being clearly covered in feathers.

Confuciusornis

STM 13-55
LSF reveals soft tissues shrouding the metatarsus and digits I and II of STM 13-55. Faint remnants of soft tissues can also be made out on the remaining digits and the digits of the right foot, although these appear to have been overprepared and no longer show interpretable details. On the left foot, crescentic patterns in the integument lateral to the proximal end of metatarsal IV may represent scutate scales based on their relatively large size and transverse arrangement.

Supplementary Descriptions of Extreme Points in PCA

The two most positive points on PC1 are the western osprey *Pandion haliaetus* and Ross's turaco *Musophaga rossae*. These taxa have the most recurved claws in the dataset, the only two with average claw curvature above 120°. The western osprey's claws are all roughly of the same curvature, while Ross's turaco has a digit II claw that is less curved than the other claws. Ospreys are well-documented as using their recurved claws to pierce into captured fish and retain a grip[5,6], whereas the curved claws of Ross's turaco likely reflect its highly arboreal lifestyle[7].

The most negative point on PC1 is the Eurasian skylark *Alauda arvensis*, whose claws are almost completely straight. The taxon is the most cursorial of those studied and inhabits mostly flatland[8]. Long straight claws likely allow the skylark to place a broader surface to the ground during running[9].

The most positive point on PC2 is the collared forest-falcon *Micrastur semitorquatus*. It has the second highest digit I/digit III claw size ratio in the dataset after the Eurasian skylark *Alauda arvensis* (larks in general have large, straight digit I claws which flatten the foot for cursorial use[9]), and the second highest digit II/digit III claw size ratio after *Microraptor* specimen STM 5-109. The collared forest-falcon seems particularly adept at taking large prey, with 12% of prey hunted in one study area being heavier than the falcon taking it[10]. In comparison, the golden eagle *Aquila chrysaetos* rarely feeds on prey more than half its body mass and feeding events involving larger prey are usually to be considered scavenging[11,12]. Other raptors with preference for hunting prey larger than themselves like the harpy eagle *Harpia harpyja*[13] are of interest for future studies to see if their digit I and II claws are similarly hypertrophied.

The most negative points on PC2 are the shrikes (Laniidae) included in this study. The digit III is unusually large in shrikes and likely performs the same pinning role as digit II in accipiters. Elaboration on this point is given by Miller et al.[14], particularly their figure S6 which shows shrikes clustering with restraint raptors when digit IV is used as a reference instead of digit III. The most negative point aside from shrikes is the sandhill crane *Antigone canadensis*, whose enlarged digit III claw likely reflects the use of kicking in aggressive displays[15] where digit III is the most likely to make contact.

Supplementary Information on Tarsometatarsus Length as a Variable for Traditional Morphometrics

Tarsometatarsus length was investigated as a potential variable for use in the traditional morphometric analysis, as it has previously been found to discriminate between raptorial[16,17] and locomotor[18] styles in modern birds. However, we found it to be problematic as a variable. Chiefly, this is because it has a high value of Blomberg's K[19] (1.5), meaning tarsometatarsus

length is much more similar among closely-related birds than under a Brownian motion model. This is consistent with past work on avian limb proportions[20]. The tarsometatarsus has been interpreted in the past as having a strong effect on the bauplan of developing birds[21], and so we interpret the strong phylogenetic signal of tarsometatarsus length as a developmental constraint. We discard tarsometatarsus length as a variable because early fliers in this study are outside crown birds (Aves) so were not necessarily under the same developmental constraints.

Nevertheless, for completeness, we find that the inclusion of tarsometatarsus length has little effect on the results. It slightly increases the accuracy of LDA analyses (an increase in Fleiss' Kappa from 0.04-0.08 depending on the method of scaling). K_{mult} and phylogenetic HSD results were not noticeably affected. Tarsometatarsus length had similar weighting in PCA to interdigital size variation, increasing with an increase in PC2. Variance explained by PCA plots was reduced (6-8% reduction in variance explained by the first two PCs). Modern bird trends changed little with the inclusion of tarsometatarsus length, with fossil taxa plotting in more positive regions of PC2.

Supplementary References

1 Tsang, L. R., Wilson, L. A. & McDonald, P. G. Comparing the toepads of Australian diurnal and nocturnal raptors with nonpredatory taxa: insights into functional morphology. *Journal of Morphology* **280**, 1682-1692 (2019).

2 Xu, X. & Zhang, F. A new maniraptoran dinosaur from China with long feathers on the metatarsus. *Naturwissenschaften* **92**, 173-177 (2005).

3 Li, Q. G. *et al.* Reconstruction of *Microraptor* and the evolution of iridescent plumage. *Science* **335**, 1215-1219, doi:10.1126/science.1213780 (2012).

4 Pu, H. Y. *et al.* A new juvenile specimen of *Sapeornis* (Pygostylia: Aves) from the Lower Cretaceous of Northeast China and allometric scaling of this basal bird. *Paleontological Research* **17**, 27-38 (2013).

5 Fowler, D. W., Freedman, E. A. & Scannella, J. B. Predatory functional morphology in raptors: interdigital variation in talon size is related to prey restraint and immobilisation technique. *PLoS ONE* **4**, e7999, doi:10.1371/journal.pone.0007999 (2009).

6 Sustaita, D., Gloumakov, Y., Tsang, L. R. & Dollar, A. M. Behavioral correlates of semi-zygodactyly in Ospreys (*Pandion haliaetus*) based on analysis of internet images. *PeerJ* **7**, e6243, doi:10.7717/peerj.6243 (2019).

7 Turner, D. A., Kirwan, G. M. & Boesman, P. F. D. in *Birds of the World* (eds J. del Hoyo *et al.*) (Cornell Lab of Ornithology, 2021).

8 Campbell, R. W., Van Damme, L. M., Johnson, S. R., Donald, P. & Garcia, E. F. J. in *Birds of the World* (ed S. M. Billerman) (Cornell Lab of Ornithology, 2020).

9 Pike, A. V. L. & Maitland, D. P. Scaling of bird claws. *J. Zool.* **262**, 73-81, doi:10.1017/s0952836903004382 (2004).

10 Rocha, A., Borges, S. H., Ovalle, J. M. R. & Barnett, A. A. Collared Forest-Falcon (*Micrastur semitorquatus*) preying on a squirrel in a fragment of Atlantic Forest with a revision of the predation events for the species. *Rev Bras Ornitol* **25**, 248-253 (2017).

11 Bedrosian, G. *et al.* Spatial and temporal patterns in golden eagle diets in the western United States, with implications for conservation planning. *J Raptor Res* **51**, 347-367 (2017).

12 Katzner, T. E. *et al.* in *Birds of the World* (eds P. G. Rodewald & B. K. Keeney) (Cornell Lab of Ornithology, 2020).

13 Aguiar-Silva, F. H., Sanaiotti, T. M. & Luz, B. B. Food habits of the harpy eagle, a top predator from the Amazonian rainforest canopy. *J Raptor Res* **48**, 24-35 (2014).
14 Miller, C. V., Pittman, M., Wang, X., Zheng, X. & Bright, J. A. Diet of Mesozoic toothed birds (Longipterygidae) from quantitative analysis of extant avian diet proxies. *BMC Biol* (In Review).
15 Tacha, T. C. Social organization of sandhill cranes from midcontinental North America. *Wildl Monogr* **99**, 3-37 (1988).
16 Ward, A. B., Weigl, P. D. & Conroy, R. M. Functional morphology of raptor hindlimbs: implications for resource partitioning. *Auk* **119**, 1052-1063, doi:10.1642/0004-8038(2002)119[1052:Fmorhi]2.0.Co;2 (2002).
17 Einoder, L. D. & Richardson, A. M. M. Aspects of the hindlimb morphology of some Australian birds of prey: a comparative and quantitative study. *Auk* **124**, 773-788, doi:10.1642/0004-8038(2007)124[773:Aothmo]2.0.Co;2 (2007).
18 Zeffer, A., Johansson, L. C. & Marmebro, A. Functional correlation between habitat use and leg morphology in birds (Aves). *Biol J Linn Soc* **79**, 461-484, doi:10.1046/j.1095-8312.2003.00200.x (2003).
19 Blomberg, S. P., Garland, T. & Ives, A. R. Testing for phylogenetic signal in comparative data: behavioral traits are more labile. *Evolution* **57**, 717-745, doi:10.1111/j.0014-3820.2003.tb00285.x (2003).
20 Bell, A. *et al.* Quantitative analysis of morphometric data of pre-modern birds: phylogenetic versus ecologic signal. *Frontiers in Earth Science* **9**, 534 (2021).
21 Bohmer, C., Plateau, O., Cornette, R. & Abourachid, A. Correlated evolution of neck length and leg length in birds. *R Soc Open Sci* **6**, doi:10.1098/rsos.181588 (2019).
