# Supplementary material for: Exceptional preservation and foot structure reveal ecological transitions and lifestyles of early theropod flyers
Source: Nat Commun. 2022 Dec 20;13:7684. doi: 10.1038/s41467-022-35039-1 (PMC9768147; doi:10.1038/s41467-022-35039-1)
Supplement: Supplementary file 3 — Description of Additional Supplementary Files [file 41467_2022_35039_MOESM3_ESM.pdf]

Description of Additional Supplementary Files

Supplementary Data 1 is an .xlsx file with three separate tabs. Tab 1 is the study's modern avian toe pad and foot scale data. Tab 2 is the study's traditional morphometric fossil claw measurements. Tab 3 is the study's entire traditional morphometric claw dataset for both modern and fossil taxa in a format that is ready for analysis.
