# Supplementary material for: Exceptional preservation and foot structure reveal ecological transitions and lifestyles of early theropod flyers
Source: Nat Commun. 2022 Dec 20;13:7684. doi: 10.1038/s41467-022-35039-1 (PMC9768147; doi:10.1038/s41467-022-35039-1)
Supplement: Supplementary file 5 — Reporting Summary [file 41467_2022_35039_MOESM5_ESM.pdf]

nature portfolio

Corresponding author(s): Michael Pittman & Xiaoli Wang

Last updated by author(s): Nov 1, 2022

Reporting Summary

Nature Portfolio wishes to improve the reproducibility of the work that we publish. This form provides structure for consistency and transparency in reporting. For further information on Nature Portfolio policies, see our Editorial Policies and the Editorial Policy Checklist.

Statistics

For all statistical analyses, confirm that the following items are present in the figure legend, table legend, main text, or Methods section.

n/a	Confirmed	
☐	☒	The exact sample size (*n*) for each experimental group/condition, given as a discrete number and unit of measurement
☐	☒	A statement on whether measurements were taken from distinct samples or whether the same sample was measured repeatedly
☐	☒	The statistical test(s) used AND whether they are one- or two-sided<br>*Only common tests should be described solely by name; describe more complex techniques in the Methods section.*
☒	☐	A description of all covariates tested
☐	☒	A description of any assumptions or corrections, such as tests of normality and adjustment for multiple comparisons
☐	☒	A full description of the statistical parameters including central tendency (e.g. means) or other basic estimates (e.g. regression coefficient) AND variation (e.g. standard deviation) or associated estimates of uncertainty (e.g. confidence intervals)
☐	☒	For null hypothesis testing, the test statistic (e.g. *F*, *t*, *r*) with confidence intervals, effect sizes, degrees of freedom and *P* value noted<br>*Give P values as exact values whenever suitable.*
☒	☐	For Bayesian analysis, information on the choice of priors and Markov chain Monte Carlo settings
☒	☐	For hierarchical and complex designs, identification of the appropriate level for tests and full reporting of outcomes
☒	☐	Estimates of effect sizes (e.g. Cohen's *d*, Pearson's *r*), indicating how they were calculated

Our web collection on statistics for biologists contains articles on many of the points above.

Software and code

Policy information about availability of computer code

Data collection	CorelDraw X8 measure tools were used to measure scaled photographs when measurements could not be taken in person.
Data analysis	All statistical analysis was done in the open source programming language R version 4.1.2, specifically the R package RRPP49 version 1.1.2.

For manuscripts utilizing custom algorithms or software that are central to the research but not yet described in published literature, software must be made available to editors and reviewers. We strongly encourage code deposition in a community repository (e.g. GitHub). See the Nature Portfolio guidelines for submitting code & software for further information.

Data

Policy information about availability of data

All manuscripts must include a data availability statement. This statement should provide the following information, where applicable:

- Accession codes, unique identifiers, or web links for publicly available datasets
- A description of any restrictions on data availability
- For clinical datasets or third party data, please ensure that the statement adheres to our policy

The images and all other data pertinent to this research are available in the main text and Supplementary Information. Source data are provided with this paper. These data can also be obtained from the corresponding authors M. P. (mpittman@cuhk.edu.hk) and X.L.W. (wangxiaoli@lyu.edu.cn). The fossil specimens investigated are available for scientific study by qualified researchers at the Carnegie Museum of Natural History (Pittsburgh, United States), Florida Museum of Natural History (Gainesville, United States), Henan Geological Museum (Zhengzhou, China), Institute of Vertebrate Paleontology & Paleoanthroplogy (Beijing, China), Museum für Naturkunde Berlin (Berlin, Germany), Shandong Tianyu Museum of Nature (Pingyi China) and the Wyoming Dinosaur Center (Thermopolis, United States). Supplementary Data 1 comprises of modern avian toe pad and foot scale data as well as traditional morphometric claw data for modern birds and early theropod flyers.

Field-specific reporting

Please select the one below that is the best fit for your research. If you are not sure, read the appropriate sections before making your selection.

☐ Life sciences ☐ Behavioural & social sciences ☒ Ecological, evolutionary & environmental sciences

For a reference copy of the document with all sections, see nature.com/documents/nr-reporting-summary-flat.pdf

Ecological, evolutionary & environmental sciences study design

All studies must disclose on these points even when the disclosure is negative.

Study description	This study presents additional evidence from the fossil feet of early theropod flyers comprising soft tissue toe pads and foot scales as well as evidence from their claws and joints to inform their grasping ability, cursoriality and feeding mode. The study interprets these foot data in the context of existing lines of ecological evidence - namely anatomy, diet, aerial and terrestrial locomotion capabilities and the environments and climates they lived in - to improve our understanding of the evolutionary ecology of early theropod flyers.
Research sample	Over 1000 early paravian fossils from the Shandong Tianyu Museum of Nature (Pingyi, China) were imaged using Laser-Stimulated Fluorescence. 12 specimens were chosen for analysis because they preserved articulated feet with exceptionally detailed toe pad and foot scale soft tissues. This involved studying the arrangement and proportions of the toe pads and foot scales, the proportions and geometry of the pedal phalanges and claws and the hinging of the foot joints. Previously-published specimens of the early birds Confuciusornis and Sapeornis with preserved foot pads were also incorporated into the study, and claw measurements from an additional specimen of the early bird Yanornis (IVPP V13558) were sourced from the literature. Claw measurements were included from published photos of Ambopteryx and Fortunguavis in order to more broadly comment on the ecology of theropod flight evolution. For the same reason, we also included photos of the Berlin and Thermopolis specimens of Archaeopteryx taken by M.P. and T.G.K.<br><br>Podothecae of 15 modern bird of prey species spanning Pandionidae, Accipitridae, Tytonidae, Strigidae and Falconidae as well as an additional sample of 21 species of modern non-predatory birds spanning Psittaciformes, Passeriformes, Caprimulgiformes and Coraciformes were studied in the collections of the University of New England Natural History Museum (Armidale, Australia) and the Ornithology Collection of the Australian Museum Research Institute (Sydney, Australia). Measurements and photographs were collected in person at the University of New England by P.R.B. and N.J.E. and at the Australian Museum by L.R.T.<br><br>Claws from 61 taxa covering a wide variety of crown bird families, particularly from raptorial birds, were sampled from the skeletal collections of the Carnegie Museum of Natural History (Pittsburgh, PA, United States) and the Florida Museum of Natural History (Gainesville, FL, United States). Measurements and photographs were collected in person by C.V.M.
Sampling strategy	The main fossil dataset derives from laser-stimulated fluorescence (LSF) imaging of over 1000 early paravian fossils from the Shandong Tianyu Museum of Nature (Pingyi, China). From this initial survey we identified 12 samples for analysis that had articulated feet with exceptionally detailed toe pad and foot scale soft tissues. These 12 species broadly sampled the phylogenetic range of early theropod flyers - including non-bird flyers, early long-tailed flying birds and early short-tailed flying birds - permitting us to conduct the study with a sufficient sample. Other fossil data from published photos were used because they were already available or because they come from single specimen taxa (Ambopteryx and Fortunguavis). First-hand collected photos from the Berlin and Thermopolis Archaeopteryx were used as they represent exemplars for the measurements required for this genus.<br><br>Modern bird specimens were checked for pathologies and sampled if they were absent. Specimens were sourced from specialist collections: University of New England Natural History Museum (Armidale, Australia), Australian Museum Research Institute (Sydney, Australia), Carnegie Museum of Natural History (Pittsburgh, PA, United States) and Florida Museum of Natural History (Gainesville, FL, United States).
Data collection	LSF data were collected first-hand at the Shandong Tianyu Museum (Pingyi, China) by M.P., T.G.K., X.L.W. and X.T.Z. Podotheca data were collected first-hand by L.R.T., P.R.B. and N.J.E at the University of New England Natural History Museum (Armidale, Australia) and Australian Museum Research Institute (Sydney, Australia). Claw data was collected first-hand by C.V.M. at the Carnegie Museum of Natural History (Pittsburgh, United States) and Florida Museum of Natural History (Gainesville, United States). Claw measurements were taken in CorelDraw by M.L. and C.V.M.. Y.T.T. and C.V.M. collected foot joint data.
Timing and spatial scale	Toe pad, foot scale, claw and joint data were collected, processed and analysed from May - June 2014, March - June 2017, April-July 2018 and Sept 2021 - Feb 2022.
Data exclusions	No data were excluded from analyses.
Reproducibility	Measurements were repeated as a test more than once by different co-authors to ensure high data quality (all attempts at replication were successful) before the data was collected. Measurement landmarks are clear and specimen numbers are associated with all samples.
Randomization	The sampling rationale resulted in 12 fossil specimens from an initial pool of over 1000 specimens, providing randomisation.
Blinding	Authors analysed the study data blindly without know what the theropod feet would show, giving blinding.

Did the study involve field work? ☐ Yes ☒ No

Reporting for specific materials, systems and methods

We require information from authors about some types of materials, experimental systems and methods used in many studies. Here, indicate whether each material, system or method listed is relevant to your study. If you are not sure if a list item applies to your research, read the appropriate section before selecting a response.

Materials & experimental systems

n/a	Involved in the study
☒	☐ Antibodies
☒	☐ Eukaryotic cell lines
☐	☒ Palaeontology and archaeology
☐	☒ Animals and other organisms
☒	☐ Human research participants
☒	☐ Clinical data
☒	☐ Dual use research of concern

Methods

n/a	Involved in the study
☒	☐ ChIP-seq
☒	☐ Flow cytometry
☒	☐ MRI-based neuroimaging

Palaeontology and Archaeology

Specimen provenance	All specimens were accessioned in an international collection and available to qualified researchers for study (Shandong Tianyu Museum, Pingyi, China). These fossils are from the Early Cretaceous Jehol Group of Liaoning, China. No permits were necessary for the work as the specimens were already in the museum's collection.
Specimen deposition	All specimens are accessioned in an international collection and available to qualified researchers for study (Shandong Tianyu Museum, Pingyi, China).
Dating methods	Geological dates come from the literature only. No new geological dates are provided.

☒ Tick this box to confirm that the raw and calibrated dates are available in the paper or in Supplementary Information.

Ethics oversight	This study follows the standards expected of professional international palaeontological societies, including the Society of Vertebrate Paleontology (USA).

Note that full information on the approval of the study protocol must also be provided in the manuscript.

Animals and other organisms

Policy information about studies involving animals; ARRIVE guidelines recommended for reporting animal research

Laboratory animals	N/A
Wild animals	N/A
Field-collected samples	Modern samples were studied in University of New England Natural History Museum (Armidale, Australia), Australian Museum Research Institute (Sydney, Australia), Carnegie Museum of Natural History (Pittsburgh, PA, United States) and Florida Museum of Natural History (Gainesville, FL, United States). These collections keep their specimens under standard conditions. Specimen were studied with standard procedures and followed the guidance of the host collections.
Ethics oversight	The collections visited (University of New England Natural History Museum, Armidale, Australia; Australian Museum Research Institute, Sydney, Australia; Carnegie Museum of Natural History, Pittsburgh, United States; Florida Museum of Natural History, Gainesville, United States) made specimens available for study according to their own ethical guidelines. These specimens were studied according to the ethical guidelines of these collections.

Note that full information on the approval of the study protocol must also be provided in the manuscript.
